# The effect of the hospital working environment on the work limitation of the employees in Turkey: a multivariable analysis

**DOI:** 10.1186/s12913-023-09356-0

**Published:** 2023-04-13

**Authors:** Mahmut Kiliç, Güllü Uslukiliç, Sevda Yaman

**Affiliations:** 1 Faculty of Medicine Department of Public Health, Yozgat Bozok University, Yozgat, Turkey; 2 Health Practice and Research Center Hospital, Yozgat Bozok University, Yozgat, Turkey; 3 Akdagmadeni Health School, Yozgat Bozok University, Yozgat, Turkey

**Keywords:** Work, Limitation, Workplace, Occupational health, Hospital Personnel

## Abstract

**Background:**

The aim of this study is to examine the effects of working environment and demographic variables on the level of work limitation in a university hospital.

**Methods:**

The study is cross-sectional and was conducted in 2022 among employees of a university hospital. 254 people voluntarily participated in the study. Data were collected by applying the sociodemographic data form, the Work Limitation Questionnaire (WLQ), and the Work Environment Scale (WES). Institutional permission and ethical approval were obtained for the study. In the analysis of the data, t-test, ANOVA, and linear regression (LR) were used.

**Results:**

The WLQ score average of hospital staff was low. According to LR analysis, the factors affecting the level of work limitation of hospital staff; worsening perception of health status, being a doctor, decreased income level, increased working time in the institution, and age reduction. It was determined that 32.8% of the change in the WLQ score was related to these factors. While in the univariate tests, the mean of work limitation was found to be significant by getting occupational health safety training, having health problems due to the work done, and taking leave due to work accidents, in the multivariable LR analysis, these factors were insignificant.

**Conclusions:**

As the working environment gets worse, the level of work limitation increases. It is recommended that hospital managers make the working environment better and safer, and make arrangements and programs to increase personnel satisfaction.

## Introduction

Working life is an indispensable part of life and covers a large part of daily life. The work environment can have many positive and negative effects on both employees and service recipients [1]. Tertiary education and research hospitals, which are classified as very dangerous according to the legislation of our country, threaten the health of workers in terms of occupational diseases and work accidents [1, 2]. Situations such as difficult working conditions, patient transport and positioning, transmission of infection, physical assault, psychological pressure and shift work put employees’ performance, relationships, productivity, and health at risk [1]. All these risk factors and situations can cause work limitations in the hospital’s employees. Work limitations that may occur due to these risks may also reduce service efficiency. All kinds of possible disruptions in health services make aggrieve to the patients, employees, managers and policy makers [3].

Few studies have been found in the literature on work limitations in hospital’s workers. However, the World Health Organization has accepted many serious and potentially fatal diseases as a risk for health workers. While the probability of experiencing health problems related to the working environment is higher in health workers than in other occupational groups, it has been found that their performance is lower [4]. In addition, work limitation is not just a concept with a health dimension, it also cares about the social consequences of disruptions at work. The Work limitation Questionnaire used in this regard represents a tool that measures the degree of interference of health problems with the ability to perform tasks at work and the productivity of the individual, and its dimensions include the multidimensional character of the functions developed at work and can explain in which areas the functions are limited [5]. All kinds of factors that may cause disruption of health services are considered by the World Health Organization [6].

The term “limitations” is used to encompass both activity limitations and participation restrictions, as conceptualized in the International Classification of Functioning (ICF) [7]. That is, it refers to both difficulties in performing a particular task or action (activity limitations) and difficulties in participating in work (participation restrictions). However, in the literature, the concept of work limitation has been emphasized on people with a specific chronic disease [8].

No study has been found on the effect of the workplace environment on the work restriction of employees who do not have physical or mental disabilities. Our research is important in terms of comprehensively addressing the problems experienced by healthcare professionals regarding their working environments. [7].

Our study examined the factors related to working limitations because of environment in the hospital’s employees with the multivariable analysis method, and evaluated the effect of social demographic characteristics of employees. We think that our research can create awareness about job limitations in healthcare workers. In addition, the results of the study can also provide evidence for the obligation of professionals responsible for employee health to ensure that employees’ work environment is healthy and safe.

## Materials and methods

### Type of research

This research is a cross-sectional type.

### Population and sample

The population of the research consists of the employees at the Yozgat Bozok Research and Application Center Hospital in Turkey in May-August 2022. The minimum sample size for the research was calculated with the GPower 3.1 program. In order to perform a linear regression analysis in which 5 factors such as work environment scale score, working time in the institution, age, occupation and gender affecting the level of work limitation of hospital employees were taken, the effect size was R2 = 0.15, type-I error α = 0.05 and 1- At β = 0.95 power, the minimum sample size was calculated as N = 138. After the necessary explanations were made, 254 people whose verbal consents were taken participated in the study.

### Data collection tools

The data were collected with the questionnaires that were filled out by hospital staff.

#### Sociodemographic questionnaire form

It consists of 17 questions created by researchers to determine variables such as age, gender, economic status, occupation, marital status, health status, occupational health and safety education, exposure to work accident.

#### Work limitation questionnaire (WLQ)-short form (WLQ-SF)

The Turkish validity and reliability of the short form of the scale, which was formed by Debra Learner (2001) in WLQ-SF long form and 25 items, was conducted by Şahin in 2019. In the short form created by Şahin, it consists of 8 questions belonging to 4 basic areas. 4 basic areas are time management, physical, mental-interpersonal relationship and productivity. The questionnaire was evaluated a 5-point Likert scale. It is evaluated in the range of always difficult (100%) never difficult (0%). Values ​​for which scale evaluation is given are summed up with their own fields in each section and their arithmetic average is taken. Each section receives a score between 0 and 100. An increase in the score indicates an increase in the limitation [9, 10]. The Cronbach value was found to be 0.83 [7, 8]. In this study, the total internal consistency of the scale was found to be Cronbach α = 0.92.

#### Work environment scale (WES)

The scale, which was translated into Turkish by Kanten in 2012, consists of 23 questions as a 5-point Likert scale. Scores between 23 and 115 are taken from the scale. An increase in the score obtained from the scale indicates that the working environment is negative and risky. The WES consisted of 6 sub scales [11]. Since the WLQ score ranges from 0 to 100, WES scores were converted to the hundred score (the score the individual received − 23/92*100) in this study in order to make it easier to understand the WES score. In this study, the total internal consistency of the scale was found to be Cronbach α = 0.93.

### Data analysis

The data were evaluated in the SPSS program. The arithmetic means of the scores were analyzed with independent t-test and ANOVA. In addition, the correlation between continuous and ordinal variables and WLQ score was examined. The WLQ scale score was taken as the dependent variable of the study, and the variables that were found to be significant from socio-demographic characteristics as independent variables and the work environment scale score were analyzed by linear regression (LR) and backward method. Categorical variables were converted into dummy variables and included in the analysis.

### Ethical permission

The ethics committee approval were obtained from Yozgat Bozok University Ethics Committee with the decision dated 20.04.2022 and numbered 32/18. Before the research, participant’s consent was obtained. The research was conducted in accordance with the principles of the Helsinki Declaration.

## Results

When the percentage distributions of sociodemographic characteristics of the participants in the study are examined; 51.2% are women, 59.4% are married, 30.3% are between the ages of 25–29, 41.3% are undergraduate graduates, 47.2% are in the nurse/midwife/ Emergency medical technician (EMT). 27.6% of participants worked in the profession for 5–9 years, 37.0% worked in this institution for 2–4 years, 48.4% had an income level between 6.000 and 7.999 TL, 50.0% had perceived their general health status as good (Table 1).

**Table 1:** Mean WES and WLQ scores of hospital staff according to their socio-demographic characteristics

				WES		t/ F*	WLQ		t/ F*
		Count	%	Mean	Sd	P	Mean	Sd	p
Gender	Female	130	51.2	44.3	18.9	0.645 0.520	38.2	21.4	0.800
	Male	124	48.8	42.7	21.8		36.0	24.0	0.425
Marriage Status	Married	151	59.4	41.3	19.7	2.092 **0.037**	35.2	21.9	1.661
	Single	103	40.6	46.7	20.9		40.0	23.7	0.98
Age Groups	20–24	44	17.3	51.8	21.0	7.904 **< 0.001**	43.5	23.9	3.997
	25–29	77	30.3	48.0	21.2		39.5	25.2	**0.004**
	30–34	54	21.3	44.3	21.7		37.8	20.5	
	35–39	40	15.7	34.5	14.6		36.3	20.5	
	40+	39	15.4	33.4	13.0		25.2	17.2	
Level of Education	High School	84	33.1	39.3	19.6	4.011 **0.008**	35.1	24.5	1.974
	Associate degree	34	13.4	43.2	21.0		37.5	22.5	0.118
	Bachelor’s degree	105	41.3	43.9	20.1		36.0	20.8	
	Master’s and above	31	12.2	53.8	19.4		46.2	22.9	
Job	Doctor	27	10.6	54.5	20.9	9.520 **< 0.001**	48.6	23.8	4.852
	Nurse-midwife-EMT	120	47.2	45.3	18.7		38.8	21.5	**0.001**
	Other health professions	21	8.3	36.4	15.4		27.7	20.2	
	Administrative staff	38	15.0	28.7	18.3		27.4	19.9	
	Auxiliary staff	48	18.9	47.7	20.8		38.5	24.7	
Profession working times	0–1 years	29	11.4	49.6	21.5	4.600 **< 0.001**	35.7	26.6	1.881
	2–4 years	69	27.2	48.9	23.2		42.2	25.1	0.114
	5–9 Years	70	27.6	44.0	19.7		38.5	20.5	
	10–14 years	57	22.4	36.6	16.6		32.5	19.9	
	15 years and above	29	11.4	36.8	13.9		32.4	21.5	
Duration working in this institution	0–1 years	61	24.0	49.4	21.1	6.730 **< 0.001**	38.3	24.8	2.505
	2–4 years	94	37.0	46.4	22.5		41.3	23.9	0.060
	5–9 Years	48	18.9	40.3	13.8		32.0	20.4	
	10 Years and above	51	20.1	34.2	16.8		33.0	18.6	
Income levels	4.500–5.999 TL	33	13.0	33.9	24.1	3.612 **0.014**	34.1	24.3	0.466
	6.000–7.999 TL	123	48.4	44.5	19.7		38.4	22.2	0.706
	8.000 -9.999 TL	54	21.3	42.9	18.4		35.3	21.8	
	10.000 TL and above	44	17.3	48.7	19.6		38.1	24.6	
Perception of health status	Moderate	85	33.5	44.6	19.4	0.758 0.470	44.4	21.7	7.170
	Good	127	50.0	43.9	19.4		34.1	21.1	**0.001**
	Very good	42	16.5	40.0	24.6		31.5	26.0	
	**Total**	**254**	**100.0**	**43.5**	**20.3**		**37.1**	**22.7**	

* The t test was used for those with two groups, and the one-way ANOVA test for those with 3 or more groups

WES: Work Environment Scale, WLQ: Work Limitation Questionnaire, EMT: Emergency medicine technician, OHS: Occupational Health and Safety

### Working environment

When the WES’s scores of the hospital staff are evaluated with the t-test or Anova test according to various characteristics of the hospital staff: The scores were higher (higher score is more negative or risky) in singles (46.7), in youngers, in high-educational level, in physicians (54.5), in less working years in the profession and working in this institution, in income level of 10.000 TL and above (48.7), and the difference was found to be statistically significant (P < 0.05). However, there was no statistically significant difference in the WES’s score mean according to gender, and perception of health status (P > 0.05) (Table 1).

**Fig. 1:**
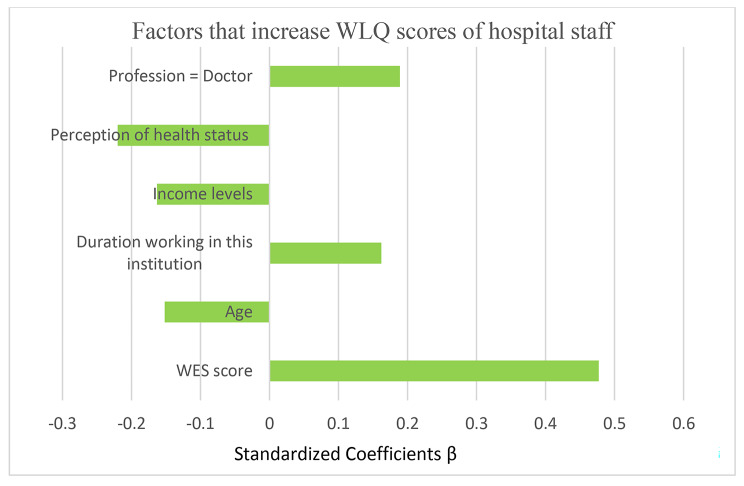
Factors that increase WLQ scores of hospital staff

When the factors that may affect the WES scores of the hospital staff were analyzed by linear regression’s backward model; most notably, an increase in the level of education (β = 0.245), a decrease in age (β= -0.236), having a health problem related to work (β = 0.227), being auxiliary staff (β = 0.152), not being administrative affairs personnel (β= -0.130), and having an occupational accident (β = 0.116) at work were found to be significant. It was determined that 26.5% (Adj.R2 = 0.265) of the change in the WES score was related to these factors. While marital status, working time in the profession and in this institution, income level and taking leave due to work accident were found to be significant in univariate tests, these variables were not found to be significant in multivariable LR analysis. In addition, the mean of WES was not found to be statistically different according to the independent variables such as gender, perception of health and OHS education (Table 2; Fig. 1).

**Table 2:** Mean WES and WLQ scores according to the problems experienced by hospital employees at work

				WES		t/ F*	WLQ		t/ F*
		Count	%	Mean	Sd	P	Mean	Sd	P
OHS training status	No	22	8.7	49.8	24.1	1.514	47.2	26.4	2.182
	Yes	232	91.3	42.9	19.9	0.131	38.2	22.3	**0.030**
Occupational accident at work	No	202	79.5	41.3	19.3	3.161 **0.002**	36.4	22.6	1.016
	Yes	52	20.5	51.9	22.2		40.0	23.1	0.313
Experiencing work-related health problems	No	168	66.1	38.5	18.6	5.691 **< 0.001**	32.9	22.0	4.282
	Yes	86	33.9	53.3	20.1		45.4	22.0	**< 0.001**
Work-related - Psychological problems	No	222	87.4	41.6	19.9	4.248 **< 0.001**	35.5	22.1	2.948
	Yes	32	12.6	56.8	18.7		48.6	23.8	**0.005**
Work-related-physical problems	No	191	75.2	40.1	19.4	4.903 **< 0.001**	35.0	22.7	2.698
	Yes	63	24.8	53.9	19.5		43.6	21.7	**0.008**
Work-related absence days	Had no problems	161	63.4	39.2	18.9	11.688 **< 0.001**	33.9	22.5	4.790
	Non	76	29.9	49.8	20.5		42.4	21.7	**0.009**
	≥ 1 Day	17	6.7	56.3	21.0		44.7	24.8	
	**Total**	254	100.0	**43.5**	**20.3**		**37.1**	**22.7**	

WES: Work Environment Scale, WLQ: Work Limitation Questionnaire, OHS: Occupational Health and Safety

### Work limitation

It was concluded that the mean of WLQ score was higher in hospital workers who were younger, doctors (48.6) and those who stated their health status as moderate (44.4), that is, the work limitation was higher (P < 0.05). WLQ score averages according to gender, marital status, education level, years of work in the profession and in this institution, and income level were not found to be statistically significant (Table 1).

When the WLQ score averages are evaluated according to the OHS status of the participants; It was determined that the work limitation was higher in those who did not receive OHS training (26.4), who had health problems due to the work done (45.4), who took one day or more leave due to work accident (44.7), and this situation was statistically significant (p < 0.05). The WLQ score mean was not found to be statistically different according to the status of receiving occupational health safety (OHS) training (Table 3).

**Table 3:** Characteristics of hospital staff and the relationship between the WES score and the WLQ score

	WLQ	WES	Age	1	2	3	4	6	7	8
**WES** ^a^	0.505^**^	1								
Age ^a^	− 0.239^**^	− 0.331^**^	1							
1.Education levels	0.104	0.184^**^	0.104	1.000						
2.Profession working times	-0.120	− 0.236^**^	0.721^**^	-0.026	1.000					
3. Duration working in this institution	-0.115	− 0.261^**^	0.654^**^	-0.065	0.763^**^	1.000				
4. Income levels	0.006	0.161^*^	0.170^**^	0.622^**^	0.170^**^	0.054	1.000			
5. Perception of health status	− 0.234^**^	-0.068	0.034	-0.085	-0.011	0.042	-0.070	1.000		
6. Receiving OHS Training	− 0.140^*^	-0.080	0.031	− 0.307^**^	0.040	0.074	− 0.298^**^	-0.025	1.000	
7. Occupational accident at work	0.055	0.192^**^	− 0.158^*^	-0.036	-0.062	-0.068	0.063	-0.046	-0.017	1.000
8. Experiencing work-related health problems	0.268^**^	0.350^**^	− 0.200^**^	0.064	-0.048	− 0.150^*^	0.034	− 0.242^**^	-0.046	0.214^**^

**. Correlation is significant at the 0.01 level (2-tailed). *. Correlation is significant at the 0.05 level (2-tailed). a Pearson correlation. 1–8. Spearman correlation was used because the variables were ordinal

WES: Work Environment Scale, WLQ: Work Limitation Questionnaire, OHS: Occupational Health and Safety

A moderately significant positive correlation was found between the WLQ and the WES (r = 0.505). There was a negative correlation between WLQ and age, health perception level, and OHS education status, but a weak positive correlation between experiencing work-related health problems. No statistically significant correlation was found between education level, work duration in the profession, occupational accident status in the institution and WLQ. A weak correlation was found between the WES score and age, working duration in the profession and working time in this institution, negatively, and positively between education level, income level, having a work accident in the institution and having work-related health problems (Table 4).

**Table 4:** Factors affecting the WES and WLQ scores of hospital staff by linear regression

	Unstandardized Coefficients		Standardized Coefficients	t	Sig.	95,0% Confidence Interval for B	
	B	Std. Error	β			Lower Bound	Upper Bound
**WES score** Adj.R^2^ = 0,265							
(Constant)	59.193	7.234		8.183	0.000	44.946	73.441
Age	− 0.613	0.147	− 0.236	-4.161	**0.000**	− 0.902	− 0.323
Education levels	4.301	1.210	0.245	3.554	**0.000**	1.918	6.684
Profession = Administrative staff	-6.796	3.183	− 0.130	-2.135	**0.034**	-13.064	− 0.527
Profession = Auxiliary staff	7.250	3.275	0.152	2.214	**0.028**	0.800	13.700
Occupational accident at work	5.363	2.576	0.116	2.082	**0.038**	0.289	10.437
Experiencing a work-related health problem	8.939	2.237	0.227	3.995	**0.000**	4.532	13.346
**WLQ score** Adj.R^2^ = 0,328							
(Constant)	40.293	10.152		3.969	0.000	20.298	60.289
WES scores	0.580	0.068	0.477	8.480	**0.000**	0.445	0.715
Age	− 0.480	0.217	− 0.152	-2.211	**0.028**	− 0.907	− 0.052
Duration working in this institution	3.488	1.489	0.162	2.342	**0.020**	0.555	6.422
Income levels	-3.999	1.559	− 0.163	-2.565	**0.011**	-7.069	− 0.929
Perception of health status	-6.483	1.531	− 0.220	-4.234	**0.000**	-9.498	-3.467
Profession = Doctor	13.935	4.804	0.189	2.901	**0.004**	4.473	23.396

WES: Work Environment Scale, WLQ: Work Limitation Questionnaire, OHS: Occupational Health and Safety

Independent variables: Continuous / ordinal: WES, Age, Education level, Years of working in the profession, Income level, Health status perception level, Dummy variables: Gender, Occupation, Marriage status, OHS training, At the institution where they work Having a work accident, Experiencing work-related health problems, Taking leave due to work-related health problems

When the factors that may affect the work limitation level of hospital staff are analyzed by linear regression’s backward model; the most important WES (β = 0.477), worsening of perception of health status (β= -0.220), being a doctor (β = 0.189) and a decrease in income level (β= -0.163), increased working duration in this institution (β = 0.162) and decreasing age (β= -0.152) were found to be significant. It was determined that 32.8% (Adj.R2 = 0.328) of the change in the WLQ score was related to these factors. While getting OHS training, having health problems due to the working, and taking leave due to work accident were found to be significant in univariate tests, these variables were not found to be significant in multivariable LR analysis. In addition, the mean of WLQ was not found to be statistically different according to the independent variables such as gender, education level, years of working in the profession, having a work accident in the institution where they work (Table 2; Fig. 1).

## Discussion

In this study, the relationship between the socio-demographic characteristics of the hospital staff, the perception of the working environment and the work limitation was examined using the multivariable analysis method. In Turkey, the inability to reach the studies on the determination of the work limitation levels of the hospital staff caused difficulties in making the discussion adequately. In addition, since the purpose of this study was not to evaluate the working environment, there was not much discussion about the results of the WES.

In our study, the factors affecting the WES scores of hospital staff were respectively; increase in education level, decrease in age, having a health problem related to the work, being a support staff, not being an administrative affairs staff, and having a work accident in the institution (Table 2; Fig. 1). In a study that determined the effect of the working environment of hospital employees on job satisfaction, it was concluded that, similar to our findings, those with a master’s degree or higher have lower perceptions of the working environment [12]. The reason for this is thought to be due to the increase in the expectations of the employees as the education level increases. Occupational accidents can negatively affect the psychological and physical health of employees [13]. It can be concluded that, depending on the accidents in the working environment, the employees directly affect their health negatively and evaluate the working environment inadequately. Occupational accidents of employees may occur due to work environment, personal reasons, administrative reasons or lack of equipment. As a result of negative experiences, the mental or physical health of the employees may be adversely affected. In our study, it was concluded that the WES score was also high in those who had work-related health problems. Many factors, such as shift work depending on the working environment, stress factor, prolonged standing work, frequent repetitive movements, caring patients with severe conditions, affect the health of employees at different levels, physically, psychologically and sociologically [14]. As a result of these work-related negativities, the risk of occupational accidents also increases [15–18]. Accordingly, it can be said that there is a positive relationship between work-related health problems and work accidents. It is expected that those who have a work-related health problem and those who have had a work accident in the institution they work for will perceive the working environment more negatively. It can be thought that people generally have a more negative attitude towards the work environment in which they are harmed, due to negative experiences.

The decrease in professional experience may be directly proportional to the decrease in age. In our study, the increase in the positive evaluation of the working environment as the profession working duration increases, explains the negative evaluation of the working environment by the younger ones. In the study among hospital employees, it was determined that participants over the age of 40 evaluated the working environment more positively than participants aged 39 and younger [12].

In our study, WLQ score averages according to gender, marital status, education level, years of employment in the profession and in this institution, and income level were not found to be statistically significant (Table 1). Contrary to our study, a research on the effect of the work environment on the mental-emotional health of healthcare professionals and their coping strategies concluded that married people generally have a more positive approach to situations, re-evaluation of problems, and problem-solving situations [19]. In this case, it is thought that being married has a positive effect on occupational health and limitations. In the same study, they concluded that long-term workers have worse work-related physical and mental health compared to new employees [19]. The reason for this difference can be thought to be due to the working conditions or the differences in the evaluation processes of the events in the country where the study was conducted.

It was concluded that the mean WLQ score was higher (increased score high limitation) among the participants in the study who were younger, doctors, and those who stated their health status as moderate (P < 0.05) (Table 1). Considering that people with work-related psychological or physical disorders are considered in the definition of work limitation, it is expected that those who state their health status as medium or low will have more work limitations.

In our study, the WES score was found to be higher in those who had an occupational accident at the institution, had health problems due to the work done, had psychological or physical problems, and who took 1 day or more leave due to an occupational accident (p < 0.05). On the working environment of the workers in the oncology hospital in Greece, it was found that the insufficiency of laminar air flow in the sections where dangerous drugs are prepared, the incorrect storage and storage of drugs, and most importantly, the absence of transportation vehicles, and the fact that the employees suffer from work accidents and occupational diseases they found that there is a relationship between [20]. It is stated that these situations are considered as hospital environment pollution and have a negative impact on the health of the employees [20, 21]. Despite the use of personal protective equipment during drug preparation by the oncology hospital staff, they reported that they complained of hair loss, acute or chronic symptoms from the CNS, respiratory, skin and musculoskeletal systems, and more rarely, intestinal, gynecological and other problems. As a result, they stated that most of the work accidents and occupational diseases were not caused by the employees not using personal protective equipment, but were caused by the working environment [20]. These studies also support our findings and prove that there is a relationship between the working environment and having a work accident and occupational disease. It is the issue that there are reciprocal relationships between work environments and work accidents, work-related health problems, work-related physical or mental problems, and being left the institution due to work accidents [22–24]. Studies show that those with a higher workload tend to report more health problems compared to those with a lower workload [25]. It can be said that it is not logically possible for employees who experience negative emotions to be satisfied with the working environment.

Increasing health and safety levels and creating a positive culture in employees will have positive results on employees’ health. This will lead to a decrease in work limitation levels. It was determined that there is a statistically significant difference between the WLQ, those who have health problems depending on the work done, and those who take one day or more leave due to work accident (Table 3). In literature, the negative effects of psychological violence arising from the working environment on the organizational climate were mentioned [26]. It increases the risk of occupational accidents and occupational diseases due to physical, psychological and behavioral reasons related to the working environment. Due to the negativities experienced, the leave situation is increasing, and in this case, it can cause negativities for the institution and the employee [26, 27]. Factors such as mobbing, lack of organizational culture and justice, and lack of healthy and safe environments affect the performance and productivity of employees. There are studies showing that these factors affect the mental health and physical health of the employees depending on the workplace and negatively affect their performance and productivity.

In our study, a moderately significant positive correlation was found between WLQ and WES (r = 0.505) (T0able 3). In the study on an emergency department employees, it has been determined that the health of the employees is adversely affected due to reasons such as physical negativities in the working environment, workload, pressure from patient relatives, and they are under more stress and experience a sense of burnout. In the literature, there are studies that support our study and conclude that the health of employees who depend on the working environment is affected [28–30].

When the factors that may affect the work limitation level of hospital staff are analyzed by linear regression; It was found that the increase in the WES score, the worsening of the perception of health status, being doctor, the decrease in the income level, the increase in the working duration in this institution, and the decrease in the age were found to be significant, respectively (Table 2; Fig. 1).

Negative working environment affects the work limitation. In a study on intensive care nurses, it is seen that the working environment affects the health of the employees. It is seen in many studies that the increase in working conditions and the negativities in the environment negatively affect the mental and physical health of the employees [30]. When the studies examining the effects of the physical and mental health of the working environment of the hospital employees are examined, it is seen that mostly the doctors and nurses have work-related musculoskeletal disorders, psychological problems, exposure to mobbing, the feeling of burnout and consequently a decrease in work efficiency, an increase in work accidents, and a decrease in job satisfaction [15–18]. The results of other studies and our study prove that there is a positive relationship between the working environment and the work limitation levels of the employees. The healthier and safer the working environment is created, the work limitation will decrease accordingly (Positive).

Job limitation refers to the mental and physical limitations of employees depending on the job. The decrease in the perception of health status indicates that the employees perceive themselves as unhappy, physically or mentally depressed. It is an expected situation that the ILO values of the employees who have this feeling are high (negative). The hospital environment is a dangerous and risky environment for all employees. However, these risks may differ according to departments and professions. It is thought that doctors’ ILO scores are higher because doctors who are closer to patients and who have more responsibilities deal with more patients and their relatives. Some findings indicate that work-related stress increases with increasing age [31]. Increasing job limitations with decreasing age does not appear to be a normally expected situation. It is thought that this situation that seems to be a contradiction arises from the fact that young workers mostly consist of non-doctor personnel, the difficulties and inexperience of young people in accepting their profession. Considering that the number of years working in the profession increases in parallel with the increase in age, the increase in working years in the institution can also be counted among the factors that decrease the ILO score.

In the multivariable LR analysis in our study, no significant relationship was found between work limitation and gender, education level, years of work in the profession, OHS training status, having a work accident in the institution where they work, having a work-related health problem and taking leave due to a work-related health problem (Table 2). In the study on nurses, they concluded that there is no significant difference in work-related tension depending on education levels and working years in the profession [31]. In another study concluded that there is no significant difference between the education levels of the employees and the working year in the profession on job stress [32]. Contrary to our study, there are studies that concluded that the level of work stress and depression affect the working year [33].

## Conclusion and recommendations

The factors that increase the level of work limitation of the hospital staff are, respectively, the increase in negative perception of the working environment, the worsening of the perception of health status, the decrease in the level of being a doctor, the decrease in the income level, the increase in the working time and the decrease in the age. The most important factor that increases the level of work limitation of hospital employees is their negative perception of the workplace environment. The fact that the younger ones evaluate the working environment negatively and their work limitations are higher indicated dissatisfaction with the working environment in the institution. It is thought that the high level of work limitations of doctors with a high level of education is also a result of dissatisfaction with the institution and work environment.

It is recommended that hospital managers make the working environment better and safer, and make arrangements and programs to increase personnel satisfaction.

Innovative policies focusing on hiring and retention, strategies for continuing education and self-renewal, remuneration for employees, awareness programs, adequate equipment and materials, and the creation of a safe working environment can be suggested.

### Limitations of the research

Conducting the research only in a university hospital constitutes the limitation of the research.

## Data Availability

This study was prepared by using the data of the master’s thesis named “Examination of the relationship between the hospital working environment and the level of work limitation and occupational health and safety culture”. The datasets generated and/or analysed during the current study are not publicly available due to limitations of ethical approval involving the participants’ data and anonymity but are available from the corresponding author on reasonable request.

## References

[1] Fein EC (2021). Clarifying the effect of work hours on health through work-life conflict. Acad Emerg Med.

[2] Kılıç M, Üstündağ N, Öcal, Uslukılıç G (2022). The effect of nursing professional satisfaction and teamwork attitude on evidence-based nursing attitude: a multivariate analysis. Turkiye Klin J Nurs Sci.

[3] Takegami M, Yamazaki S, Greenhill A, Chang H, Fukuhara S (2014). Work performance assessed by a newly developed japanese version of the work limitation questionnaire in a general japanese adult population. J Occup Health.

[4] De Oliveira SA, Campos JADB, Marôco J, Marziale MHP, Rocha FLR. Psychometric properties of the Work Limitations Questionnaire applied to nursing workers. Rev Latino Am Enfermagem. 2021;29:e3466. 10.1590/1518-8345.4771.3466 10.1590/1518-8345.4771.3466 PMC8432506 34468623

[5] Lerner D, Amick BC, Rogers WH, Malspeis S, Bungay K, Cynn D. The Work Limitations Questionnaire. Med Care. 2001;39(1):72–85. https://www.jstor.org/stable/3767701 10.1097/00005650-200101000-00009 11176545

[6] World Health Organization., “Global Health Workforce Network,” 2017;1(May):2–3.

[7] McLeod S, Threats T (2008). The ICF-CY and children with communication disabilities. Int J Speech Lang Pathol.

[8] Theis KA, Murphy L, Hootman JM, Helmick CG, Yelin E (2007). Prevalence and correlates of arthritis-attributable work limitation in the US population among persons ages 18–64: 2002 National health interview survey data. Arthritis Care Res.

[9] Güven B. İş güvenliği ve işgören sağlığı kültürü ve örgütsel vatandaşlık davranışı, 1.Baskı. İstanbul, 2019.

[10] Şahin R (2019). İş Limitasyonu Ölçeği Kısa Formu Türkçe Uyarlaması: Geçerlilik Ve Güvenilirlik Çalışması.

[11] Kanten S. Çalışma Koşullarının Fiziksel - Psikolojik Sağlık Belirtileri ve İş Kazaları ile İlişkisi: Mermer Çalışanları Örneği. Mehmet Akif Ersoy Univ J Soc Sci Inst. 2012;4(7):155–67. 10.20875/SB.54394.

[12] Saygili M, Çelik Y. “Hastane Çalışanlarının Çalışma Ortamlarına İlişkin Algıları İle İş Doyumu Düzeyleri Arasındaki İlişkinin Değerlendirilmesi *,”Hacettepe Sağlık İdaresi Derg., 2011;14(1).

[13] Erdoğan E. İş Sağlığı ve Güvenliği Kültürünün Önemi. In: 5th international congress on political, economic and social studies (ICPESS), 2018.

[14] Bacak B, Kazancı E (2014). Türk çalışma hayatında vardiyalı gece çalışan işçilerin karşılaştığı fizyolojik, psikolojik ve sosyolojik etkilerin değerlendirilmesi. Hak İş Uluslararası Emek ve Toplum Dergisi.

[15] Tel H, Tel Aydın H, Karabay G, Günaltay H, Akay D. Hemşirelerde İşe Bağlı Gerginlik ve Stresle Başetme Durumu. Cumhuriyet Hemşirelik Dergisi, 2012.

[16] Ahmet Sunter, Canbaz S, Dabak Ş, Öz H, Pekşen Y (2006). Pratisyen hekimlerde tükenmişlik, işe bağlı gerginlik ve iş doyumu düzeyleri related papers. Genel Tıp Derg.

[17] Yıldırım D, Yıldırım A. Saglik Alaninda Çalisan Akademisyenlerin Karsilastiklari Psikolojik Siddet Davranislari ve Bu Davranislarin Etkileri. Turkiye Klinikleri J Med Sci. 2010.

[18] Parlar S (2008). Sağlık Çalışanlarında Göz Ardı Edilen Bir Durum: Sağlıklı Çalışma Ortamı. TSK koruyucu Hekim bülteni.

[19] Koinis A, Giannou V, Drantaki V, Angelaina S, Stratou E, Saridi M. The impact of Healthcare Workers Job Environment on their Mental-emotional health. Coping strategies: the case of a local General Hospital. Heal Psychol Res. Apr. 2015;3(1). 10.4081/HPR.2015.1984. 10.4081/hpr.2015.1984 PMC4768542 26973958

[20] Constantinidis TC, et al. Occupational health and safety of personnel handling chemotherapeutic agents in greek hospitals. Eur J Cancer Care (Engl). Jan. 2011;20(1):123–31. 10.1111/J.1365-2354.2009.01150.X. 10.1111/j.1365-2354.2009.01150.x 20148939

[21] McDiarmid MA, Condon M (2005). Organizational Safety Culture/Climate and Worker Compliance with Hazardous Drug Guidelines: Lessons from the blood-borne Pathogen Experience on JSTOR. J Occup Environ Med.

[22] Lockley SW, Barger LK, Ayas NT, Rothschild JM, Czeisler CA, Landrigan CP. Effects of health care provider work hours and sleep deprivation on safety and performance. Jt Comm J Qual Patient Saf. 2007;33(11):7–18. 10.1016/S1553-7250(07)33109-7. 10.1016/s1553-7250(07)33109-7 18173162

[23] Barger LK, Lockley SW, Rajaratnam SMW, Landrigan CP. Neurobehavioral, health, and safety consequences associated with shift work in safety-sensitive professions. Curr Neurol Neurosci Rep. 2009;9(2):155–64. 10.1007/s11910-009-0024-7 19268039

[24] Caruso CC et al. Long working hours, safety, and health: Toward a national research agenda. Am J Ind Med. 2006;49(11):930–42. 10.1002/AJIM.20373. 10.1002/ajim.20373 16948157

[25] Tomic W, Tomic E. Existential fulfillment and burnout among principals and teachers. J Beliefs Values. 2008;29(1):11–27. 10.1080/13617670801928191.

[26] Vartia M. “The sources of bullying–psychological work environment and organizational climate,” Eur. J. Work Organ. Psychol, Jun. 1996;5(2):203–214. 10.1080/13594329608414855.

[27] Kocabaş F, et al. Çalışma ortamında psikososyal risk etmenlerinin iş kazası, meslek hastalıkları ve işle ilgili hastalıklarla ilişkisi. Sos Güvence. no. Dec. 2018;14:28–62. 10.21441/SGUZ.2018.68.

[28] Browning L, Ryan CS, Thomas S, Greenberg M, Rolniak S. Nursing specialty and burnout. Psychol Health Med. 2007;12(2):148–54. 10.1080/13548500600568290 10.1080/13548500600568290 17365896

[29] Kebapçi A, Akyolcu N (2011). Acil birimlerde çalisan hemsirelerde çalisma ortaminin tükenmislik düzeylerine etkisi. Turkiye Acil Tip Derg.

[30] Altınöz Ü (2017). Perception of working environment, psychological distress and affecting factors in nurses working intensive care units. J Psychiatr Nurs.

[31] Arıkan D, Karabulut N, HEMŞİRELERDE İŞE BAĞLI GERGİNLİK, VE BUNU ETKİLEYEN. Anadolu Hemşirelik ve Sağlık Bilim Derg. 2004;7(1).

[32] Durmuş B, Yıldız H. Elazığ il merkezindeki hastanelerde çalışan hemşirelerin iş stres puanlarının değerlendirilmesi, VII. Ulus. Hemşirelik Kongresi Kitabı, Erzurum, pp. 280–86, 1999.

[33] Aslan SH, Gürkan BS, Girginer H, Ünal M. “İşe bağlı gerginlik ölçeğinin bir hemşire örnekleminde geçerlik ve güvenirliği,”Psikiyatri Psikoloji Psikofarmakoloji Dergisi, 1996.

